# Maintained partial protection against *Streptococcus pneumoniae* despite B‐cell depletion in mice vaccinated with a pneumococcal glycoconjugate vaccine

**DOI:** 10.1002/cti2.1366

**Published:** 2021-12-28

**Authors:** Giuseppe Ercoli, Elisa Ramos‐Sevillano, Emma Pearce, Sara Ragab, David Goldblatt, Gisbert Weckbecker, Jeremy S Brown

**Affiliations:** ^1^ Centre for Inflammation and Tissue Repair UCL Respiratory Division of Medicine University College Medical School Rayne Institute London UK; ^2^ Department of Immunobiology UCL Great Ormond Street Institute of Child Health NIHR Biomedical Research Centre London UK; ^3^ Novartis Institute for BioMedical Research Novartis Basel Switzerland

**Keywords:** B‐cell depletion, CD20, immunity, *Streptococcus pneumoniae*, vaccination

## Abstract

**Objectives:**

Anti‐CD20 monoclonal antibody therapy rapidly depletes > 95% of CD20^+^ B cells from the circulation. B‐cell depletion is an effective treatment for autoimmune disease and B‐cell malignancies but also increases the risk of respiratory tract infections. This effect on adaptive immunity could be countered by vaccination. We have used mouse models to investigate the effects of B‐cell depletion on pneumococcal vaccination, including protection against infection and timing of vaccination in relation to B‐cell depletion.

**Methods:**

C57BL/6 female mice were B‐cell depleted using anti‐CD20 antibody and immunized with two doses of Prevnar‐13 vaccine either before or after anti‐CD20 treatment. B‐cell repertoire and *Streptococcus pneumoniae*–specific IgG levels were measured using whole‐cell ELISA and flow cytometry antibody‐binding assay. Protection induced by vaccination was assessed by challenging the mice using a *S*. *pneumoniae* pneumonia model.

**Results:**

Antibody responses to *S. pneumoniae* were largely preserved in mice B‐cell depleted after vaccination resulting in full protection against pneumococcal infections. In contrast, mice vaccinated with Prevnar‐13 while B cells were depleted (with > 90% reduction in B‐cell numbers) had decreased circulating anti–*S. pneumoniae* IgG and IgM levels (measured using ELISA and flow cytometry antibody binding assays). However, some antibody responses were maintained, and, although vaccine‐induced protection against *S. pneumoniae* infection was impaired, septicaemia was still prevented in 50% of challenged mice.

**Conclusions:**

This study showed that although vaccine efficacy during periods of profound B‐cell depletion was impaired some protective efficacy was preserved, suggesting that vaccination remains beneficial.

## Graphical abstract

Timing of pneumococcal vaccination in B‐cell–depleted patients is critical for vaccine efficacy. This study shows that, although full protection can only be ensured by vaccinating mice before depletion, vaccination during/after depletion can also provide a degree of immunity against *S*. *pneumoniae*.

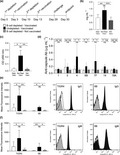

## Introduction

B cells are one of the main components of adaptive immunity playing a key role in the immune system regulation. B cells are involved in antibody production, antigen presentation and they contribute directly to inflammatory pathways.^1^, ^2^, ^3^ Dysregulation of B‐cell function can lead to the development of self‐reactive B cells resulting in autoimmune diseases such as rheumatoid arthritis, systemic lupus erythematosus, multiple sclerosis, immune thrombocytopenia^4^, ^5^ or in neoplastic conditions including lymphoma, chronic lymphocytic leukaemia and myeloma.^6^, ^7^ The use of monoclonal antibody treatment to target and selectively deplete B cells is well established in the treatment of B‐cell malignancies, and a similar approach is used in the treatment of autoimmune disorders.^8^ Several antibodies have been developed to target specific B cells subsets, among them, monoclonal antibodies against receptors CD19, CD20, and CD22.^9^, ^10^, ^11^ However, B‐cell depletion therapy has some risks, with a reported increased incidence of infections,^12^ including pneumonia and other respiratory tract infections.^13^, ^14^, ^15^, ^16^ *Streptococcus pneumoniae* (the pneumococcus) is a common cause of respiratory tract infections, and anti‐pneumococcal vaccination is routinely administered to B‐cell depleted patients to help prevent subsequent infections. The timing of vaccination is crucial, with evidence from recent publications reporting that vaccine responses can be impaired for up to 6 months after B‐cell depletion treatment.^17^, ^18^ For this reason, treatment guidelines for patients undergoing B‐cell depletion therapy recommend vaccination against major pathogens 2–3 weeks before B‐cell depletion.^19^ However, the clinical situation may prevent vaccination at the optimum time, and at present there are limited pre‐clinical data on how the timing of B‐cell depletion may affect antibody‐mediated immunity to specific pathogens. We have recently published data on the effects of B‐cell depletion in a mouse model of naturally acquired immunity to *S. pneumoniae* after nasopharyngeal colonisation,^20^ demonstrating B‐cell depletion impaired colonisation‐induced protective immunity. In this study, we have used the same mouse model to characterise the effects of timing of B‐cell depletion on immunity to *S. pneumoniae* pneumonia when vaccinated with a pneumococcal conjugated vaccine (PCV, Prevnar‐13) consisting of capsular antigen for 13 different serotypes conjugated to a carrier protein.

## Results

### Maintained antibody recognition of *S. pneumoniae* in mice vaccinated with Prevnar‐13 before B‐cell depletion

**Figure 1:**
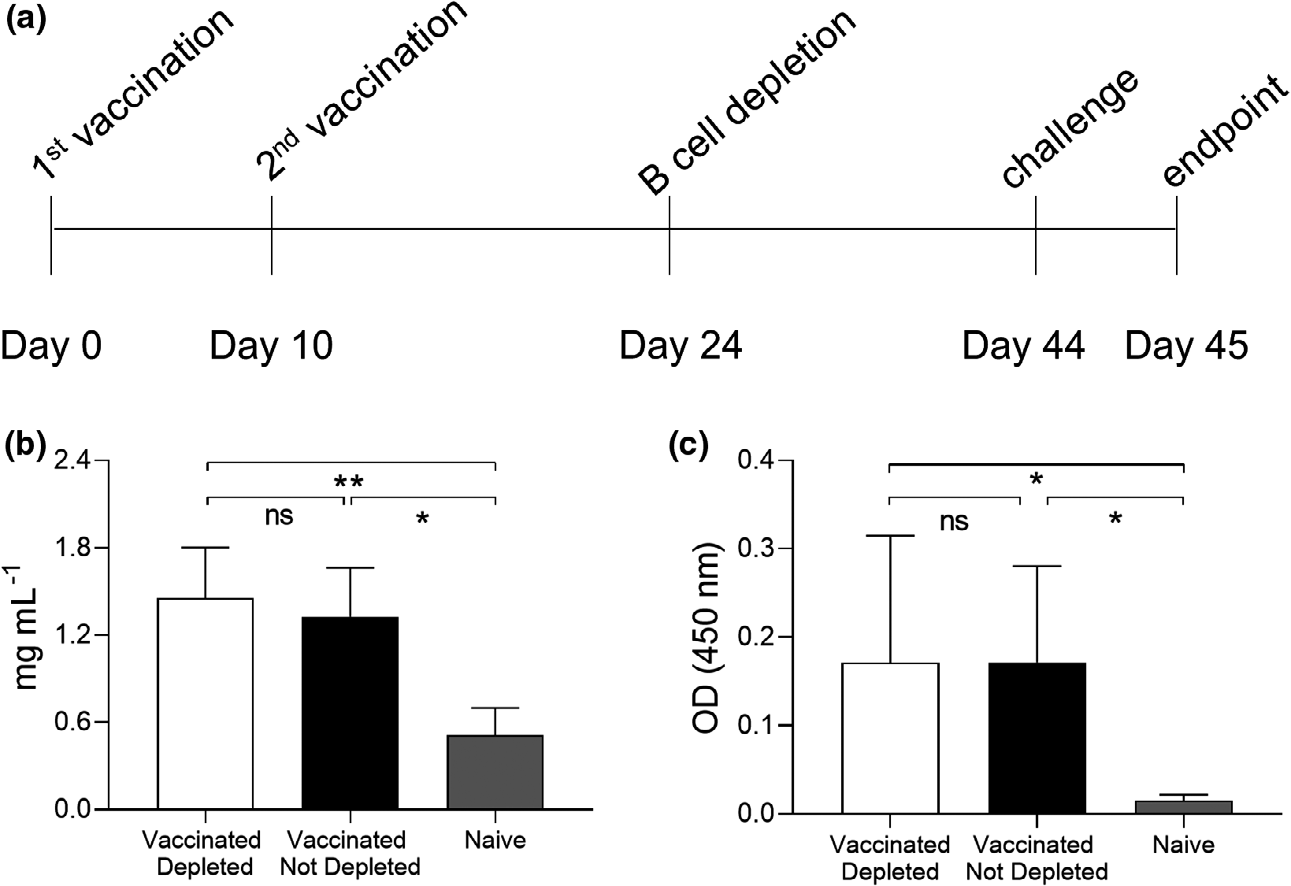
Vaccination B‐cell depletion. **(a)** Full schedule of Prevnar‐13 vaccination followed by B‐cell depletion treatment with aCD20 antibody on C57BL/6 mice. B‐cell depletion treatments were administered by intraperitoneal (i.p.) injection of 50 µg of aCD20 antibody, vaccinations by i.p. injection of 20 µL volume of Prevnar‐13 vaccine and challenge by intranasal instillation of 10^7^ pneumococcal colony forming units (CFU). A single mouse experiment was performed with mice divided into three groups depending on the treatment received: vaccinated depleted (*n* = 6), vaccinated not depleted (*n* = 6) and naive (*n* = 6). **(b)** Total IgG concentrations (mg mL^−1^) have been measured in mouse sera collected at the endpoint. **(c)** IgG specific to pneumococcus were also measured for all the mouse sera using a whole‐cell ELISA assay. In **b** and **c** **,** columns represent the average values for each group, error bars indicate Standard Deviations and the Kruskal–Wallis's multiple comparison post hoc was used for statistical analysis (ns, not significant, *P < 0.05 and **P < 0.01). All the sera were tested in triplicate and average values are reported.

We have previously established a mouse model of B‐cell depletion in which one dose (50 µg) of anti‐CD20 antibody causes over 95% reduction in spleen, lymph node and circulating B‐cell numbers.^20^ In this model, total B cells numbers were fully restored after three weeks, although reconstitution of the follicular B‐cell subset population was slightly impaired. To investigate whether established serological responses to vaccination are affected by subsequent B‐cell depletion treatment, C57BL/6 mice received two doses of Prevnar‐13 vaccine then were given one dose of B‐cell depletion therapy 14 days after the second vaccination (Figure 1a). Twenty days after depletion, at which timepoint splenic B cells would have repopulated,^20^ the mice were challenged using a *S. pneumoniae* 6B BHN418 pneumonia model. In serum obtained 21 days after B‐cell depletion total IgG levels were not affected (Figure 1b). Serum serological response to *S. pneumoniae* were measured using whole‐cell ELISA against the 6B BHN418 strain. There were no differences in ELISA IgG response between the control vaccinated and B‐cell–depleted vaccinated mice, with serum from both groups showing significant increases in pneumococcus recognition compared to sera from unvaccinated mice (Figure 1c).

**Figure 2:**
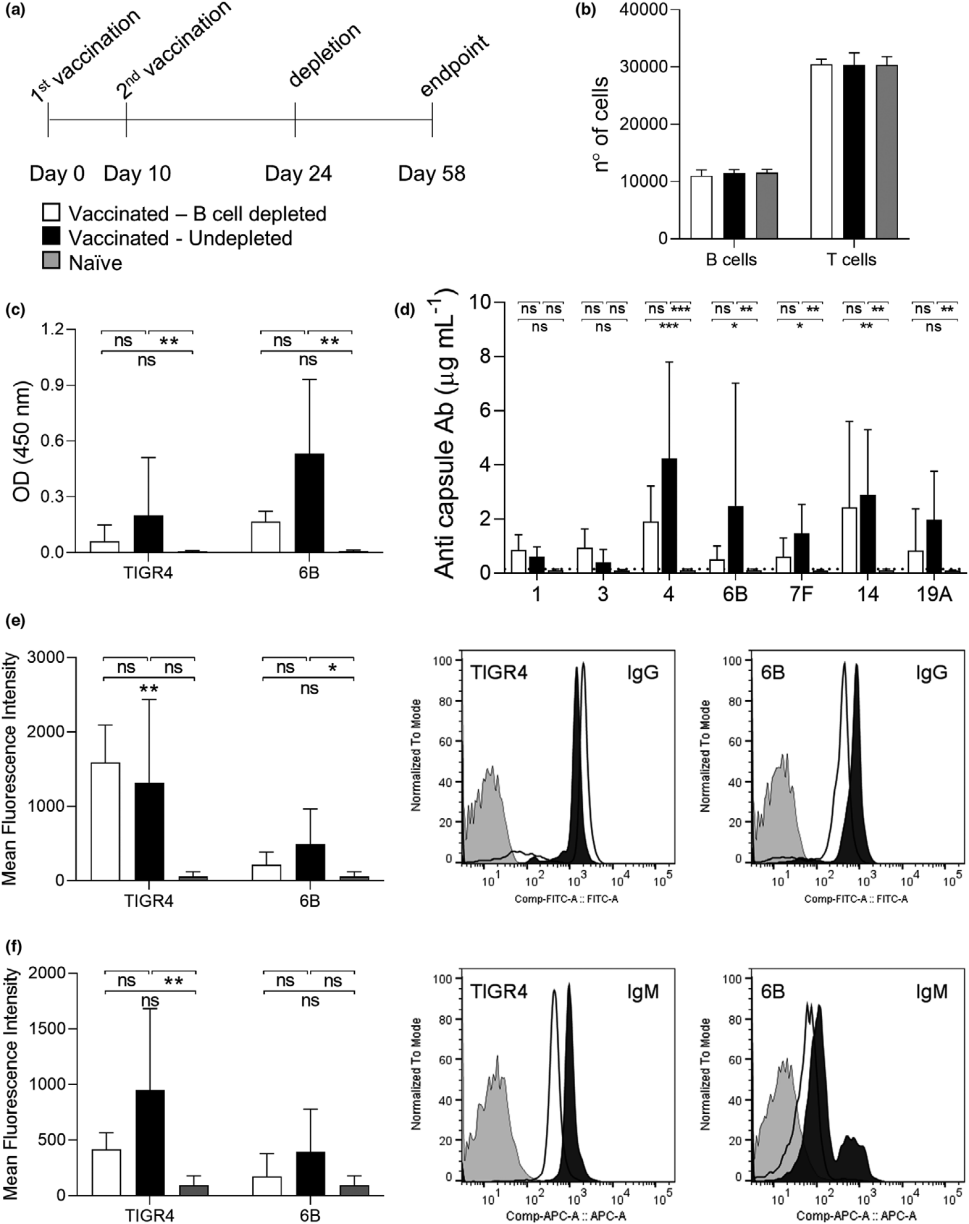
Effect of B cell reconstitution on anti‐pneumococcus antibodies. **(a)** Schedules of the depletion and vaccination treatments extended to allow B cell reconstitution are reported. A single mouse experiment was performed with mice divided into three groups depending on the treatment received: vaccinated depleted (*n* = 6), vaccinated not depleted (*n* = 6) and naive (*n* = 6) **(b)** Number of B (CD19^+^) and T cells (CD3^+^) measured by flow cytometry for 100 000 isolated splenocytes are shown. In all histograms, columns represent average values and error bars standard deviations, depleted‐vaccinated mice are shown in white, not depleted‐vaccinated in black and naïve in grey. **(c)** Mouse sera collected at the endpoint were used to measure IgG specific to TIGR4 and 6B pneumococcal strains by whole‐cell ELISA assay. **(d)** Meso Scale Discovery (MSD) assay was used to measure the specific IgG response to seven pneumococcal capsule serotypes by quantifying their concentration (µg mL^−1^). The dotted line indicates the limit of detection. **(e, f)** Serum deposition assay was used to measure the amount of surface‐exposed pneumococcal‐specific IgG **(e)** and IgM **(f)**. The average mean fluorescence intensity (MFI) for each group is reported in the left panels. Representative histograms showing a shift in populations with positive antibody binding against TIGR4 and 6B pneumococcal strains are shown in the right panels. Depleted (white), not depleted (black) and not vaccinated (grey) representative samples are shown. For all experiments, the sera were tested in triplicate and average values are reported; the Kruskal–Wallis test followed by Dunn's multiple comparison post hoc was used for statistical analysis (ns, not significant, *P < 0.05; ** and *P* < 0.01). Representative histograms showing a shift in populations positive for IgM against TIGR4 and 6B pneumococcal strains are shown in the right panels.

To further confirm that B‐cell depletion after Prevnar‐13 vaccination has little effect on antibody recognition of *S. pneumoniae*, the experiment was repeated using an endpoint 48 days after the second Prevnar‐13 vaccination equating to two IgG half‐lives^21^ (Figure 2a). Analysis of splenic lymphocytes using flow cytometry showed full reconstitution B and T cells at this timepoint (34 days post–B‐cell depletion treatment; Figure 2b). Whole‐cell ELISA against strains 6B BHN418 and TIGR4 confirmed no significant differences between depleted and undepleted vaccinated mice IgG response (Figure 2c). Anti‐capsular antibody levels to seven serotypes included in Prevnar‐13 were measured in serum from vaccinated mice and the controls using an established MSD assay.^22^ This showed no statistically significant differences in anti‐capsular antigen responses between the B‐cell–depleted and –undepleted vaccinated group for any of the serotypes tested (Figure 2d). Opsonisation with serum IgG and IgM of two different *S. pneumoniae* strains with different capsule types was assessed using an established flow cytometry assay^23^ (Figure 2e and f). The IgG data for both strains reflected the previous observations of preserved IgG recognition of *S. pneumoniae* despite B‐cell depletion (Figure 2e). The differences in binding of IgM to both *S. pneumoniae* strains in sera from B‐cell depleted or untreated vaccinated mice were also not statistically significant (Figure 2f).

### B‐cell depletion before PCV vaccination impairs vaccine‐induced antibody responses

**Figure 3:**
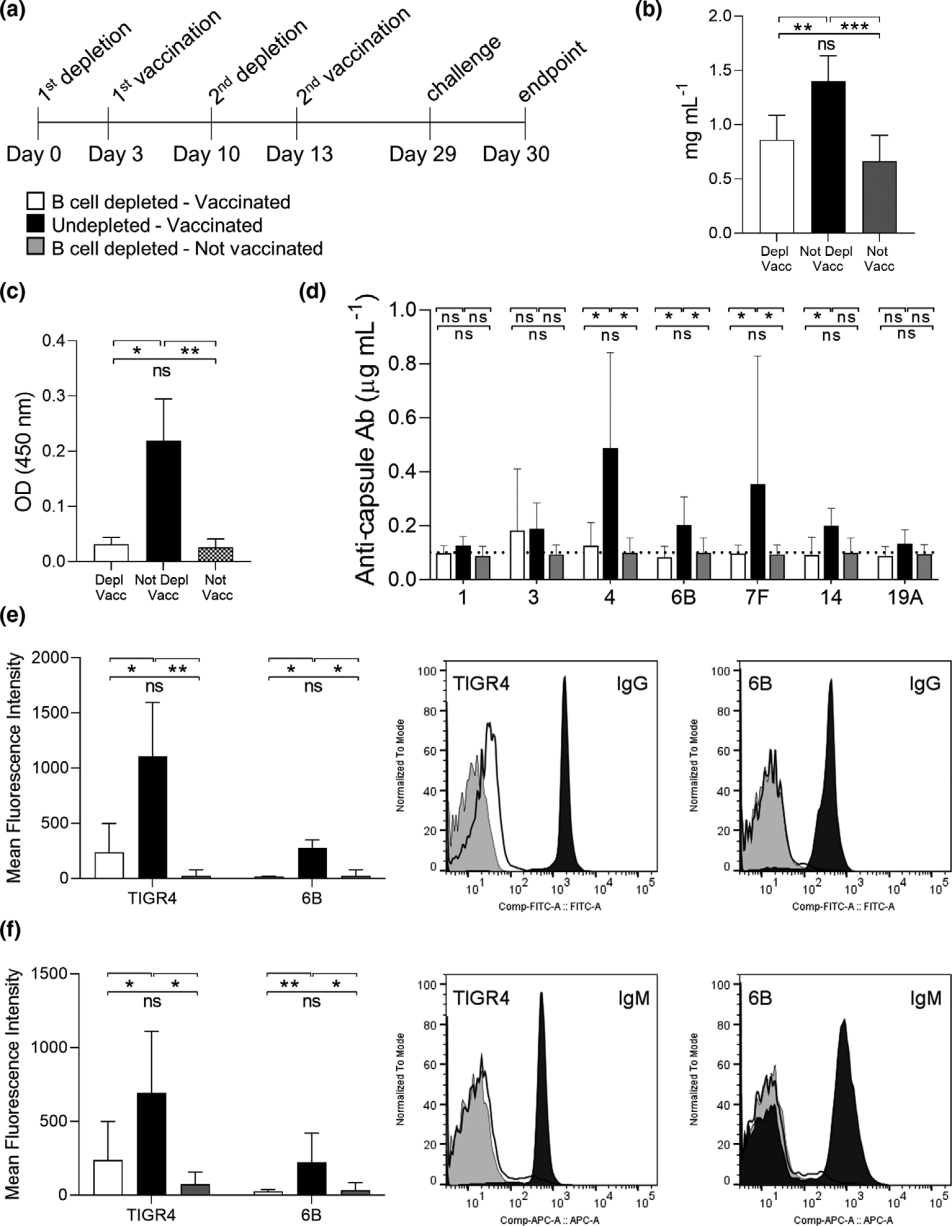
Effects of B‐cell depletion before Prevnar‐13 vaccination on serological responses. **(a)** Full schedule of B‐cell depletion treatment with aCD20 antibody followed by Prevnar‐13 vaccination on C57BL/6 mice. A single mouse experiment was performed with mice divided into three groups depending on the treatment received: depleted vaccinated (*n* = 6), not depleted vaccinated (*n* = 6) and not vaccinated (*n* = 6). **(b)** Total IgG concentrations (mg mL^−1^) in sera collected at the endpoint have been measured for each group. In all histograms, columns represent average values and error bars standard deviations, depleted‐vaccinated mice are shown in white, not depleted‐vaccinated in black and not vaccinated in grey. **(c)** IgG specific to pneumococcus have been measured for all the mouse sera using a whole‐cell ELISA assay against *Streptococcus pneumoniae* strain 6B. **(d)** The Meso Scale Discovery (MSD) assay was used to measure the specific IgG response to seven pneumococcal capsule serotypes by quantifying their concentration (µg mL^−1^). The dotted line indicates the limit of detection. **(e, f)** Serum deposition assay was used to measure the amount of surface‐exposed pneumococcal‐specific IgG **(e)** and IgM **(f)**. The average mean fluorescence intensity for each group is reported in the left panels. Representative histograms showing a shift in populations with positive antibody binding against TIGR4 and 6B pneumococcal strains are shown in the right panels. Depleted (white), not depleted (black) and not vaccinated (grey) representative samples are shown. For all experiments, the sera were tested in triplicate and average values are reported, the Kruskal–Wallis test followed by Dunn's multiple comparison post hoc was used for statistical analysis (ns, not significant, *P < 0.05; **P < 0.01 and ***P < 0.001). Representative histograms showing a shift in populations positive for IgM against TIGR4 and 6B pneumococcal strains are shown in the right panels.

To assess the effect of B‐cell depletion treatment prior to vaccination on serological responses mice were treated with B‐cell depletion therapy followed by vaccination with PCV after 3 days on two occasions separated by 7 days (Figure 3a). Under these conditions, PCV was administered to animals at a timepoint when their B cell resident population was reduced to less than 5% of the original.^20^ For the B‐cell depletion group total serum IgG level was reduced compared to vaccinated controls non–B‐cell–depleted mice but was unchanged compared to control mice total IgG (Figure 3b). B‐cell depletion just before vaccination had a profound impact on the serological responses to the PCV vaccine. When measured using ELISAs there were decreased levels of anti‐6B *S. pneumoniae* specific IgG in sera from B‐cell–depleted mice (Figure 3c). Furthermore, the MSD assay for anti‐capsular antibody showed no detectable anti‐capsular antibody in these sera, except for serotype 3, whereas sera from undepleted PCV vaccinated mice showed significant levels of IgG binding to four out of seven capsule types (serotypes 4, 6B, 7F and 14; Figure 3d). To further confirm these effects, flow cytometry was used to assess IgG and IgM opsonization to the serotype 4 and 6B strains. This assay showed reduced *S. pneumoniae* opsonization with both IgG and IgM after incubation in sera from mice that were B‐cell depleted before Prevnar‐13 vaccination compared to untreated vaccinated mice (Figure 3e and f). Overall, these data demonstrate that major B‐cell depletion at the time of PCV vaccination markedly impaired PCV‐induced IgG and IgM recognition of *S. pneumoniae*.

### Effects of B‐cell depletion on Prevnar‐13 induce protection against *S. pneumoniae* pneumonia

**Figure 4:**
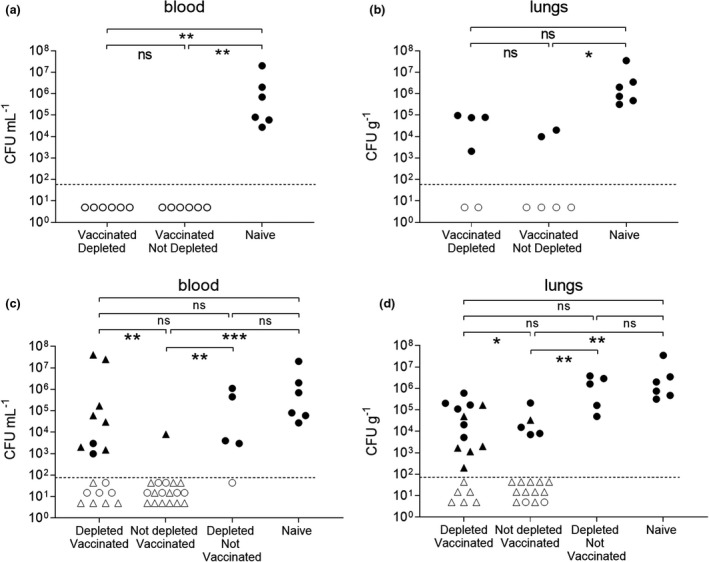
Effects of B‐cell depletion on IP Prevnar‐13 vaccination–induced protection determined after pneumonia challenge with strain *Streptococcus pneumoniae* 6B strain BHN418. In the vaccination‐depletion study pneumococcal pneumonia was induced by infecting the mice intranasally with the 6B strain at day 44. **(a, b)** A single mouse experiment was performed with mice divided into three groups depending on the treatment received: vaccinated depleted (*n* = 6), vaccinated not depleted (*n* = 6) and naive (*n* = 6). Twenty‐four hours after the infection, colony forming units (CFU) were measured from blood **(a)** and lungs **(b)**. In the depletion‐vaccination study pneumococcal pneumonia was induced by infecting the mice intranasally with 6B strain at day 29. **(c, d)** Mice were divided in 4 groups depending on the treatment received: B‐cell–depleted vaccinated (*n* = 18), not depleted vaccinated (*n* = 18) depleted not vaccinated naïve (*n* = 5) and naïve (*n* = 6). Data from two independent experiments were pooled, with experiment 1 represented by circular symbols and experiment 2 (with no control groups) triangular symbols. Twenty‐four hours after the infection, CFU were measured from blood **(c)** and lungs **(d)**. Each symbol represents the CFU value from a single mouse; mice were considered infected (black dots) when their CFU number was higher than the limit of detection (dotted line). Empty dots represent uninfected mice with 0 recovered CFU. Their position on the *Y* axis is varied to improve visual presentation and does not represent any differences in CFU. Statistical differences between groups are indicated by thin black lines (ns, not significant, *P < 0.05 and **P < 0.01 and ***P < 0.001. Fisher’s exact test, two‐tailed).

To assess the functional effects of B‐cell depletion on Prevnar‐13–induced protection against *S. pneumoniae*, Prevnar‐13–vaccinated mice with or without B‐cell depletion were challenged using an established model of serotype 6B *S. pneumoniae* pneumonia. For mice treated with B‐cell depletion after Prevnar‐13 vaccination both B‐cell depleted, and control vaccinated mice were protected against *S. pneumoniae* pneumonia (Figure 4a and b). No vaccinated mice from either group developed bacteremia (Figure 4a). In addition, lung colony forming units (CFU) were reduced for both vaccinated groups compared to the unvaccinated controls although these differences were statistically not significant due to the relative low *n* number (Figure 4b). Mice treated with B‐cell depletion before Prevnar‐13 vaccination were challenged using the *S. pneumoniae* 6B BHN418 strain pneumonia model 16 days after the second vaccination. Vaccinated mice without B‐cell depletion were almost completely protected against *S. pneumoniae* septicaemia (Figure 4c). Despite the effects of B‐cell depletion on both IgG and IgM recognition of *S. pneumoniae*, mice treated with B‐cell depletion before Prevnar‐13 vaccination still had moderate degrees of protection against *S. pneumoniae* septicaemia. The results were pooled from two separate experiments and the comparison to the combined data for unvaccinated controls (depleted not vaccinated and naïve mice) showed blood CFU only detected in 50% of B‐cell–depleted vaccinated mice compared to 91% of unvaccinated mice (*P* = 0.0436). Combined lung CFU data also showed persisting partial protection against *S. pneumoniae* pneumonia in the B‐cell depleted then vaccinated mice, with CFU detected in 67% of this group compared to 100% of unvaccinated controls (*P* = 0.0576). Overall, these data show that B‐cell depletion occurring after completion of PCV vaccination did not affect PCV‐mediated immunity to *S. pneumoniae* and that Prevnar‐13 vaccination shortly after B‐cell depletion treatment may still be able to induce partial protection against *S. pneumoniae* pneumonia.

### Vaccination route of administration has no influence on B‐cell depletion efficacy

**Figure 5:**
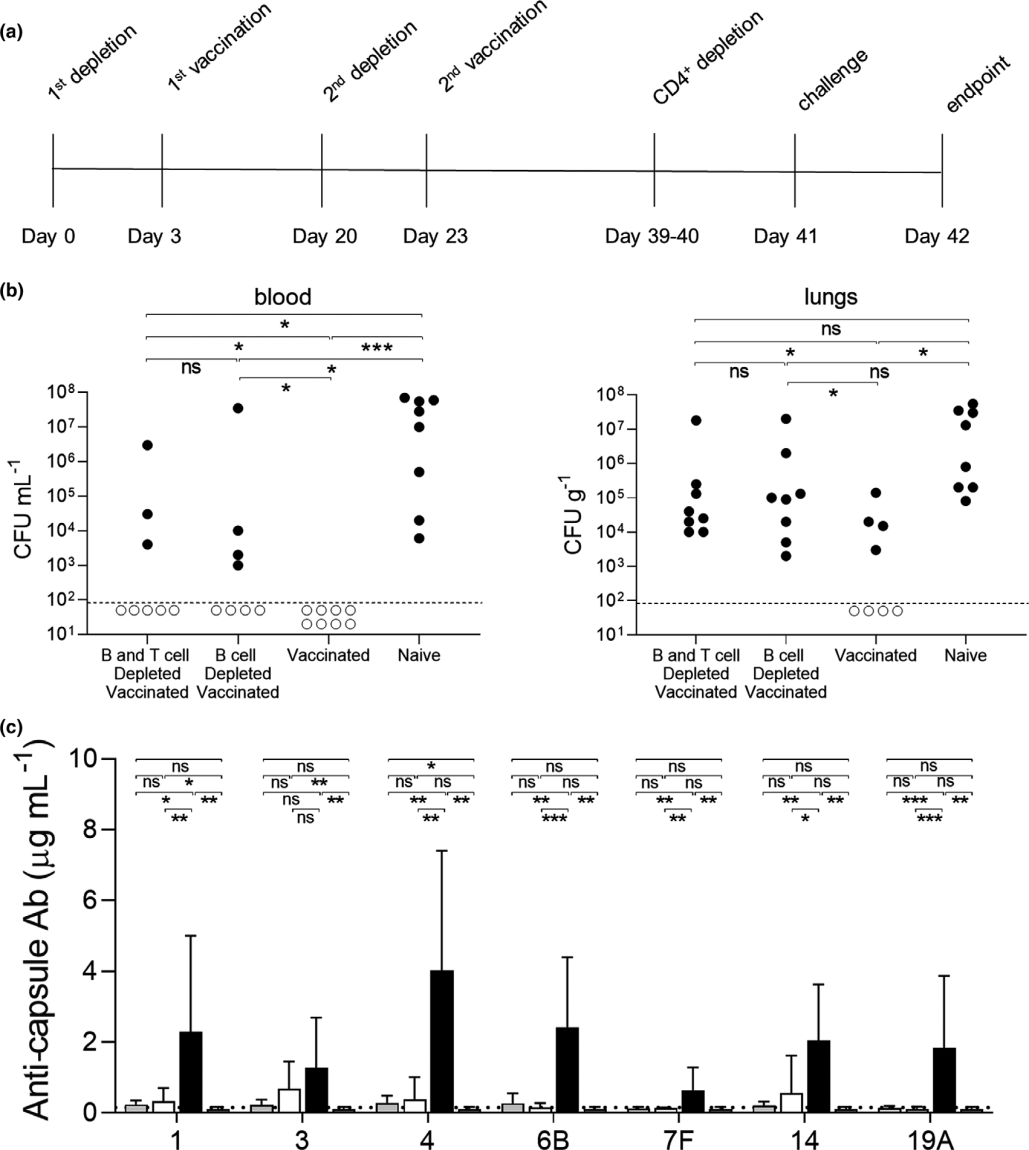
Effects of IM vaccination on protection against *Streptococcus pneumoniae* 6B strain BHN418 pneumonia challenge. **(a)** Pneumococcal pneumonia was induced infecting the mice intranasally with the 6B strain at day 41 after two rounds of intramuscular Prevnar‐13 vaccination. A single mouse experiment was performed with mice divided into four groups depending on the treatment received: B‐cell depleted then vaccinated and given additional T‐cell depletion pre‐challenge (*n* = 8), B‐cell depleted then vaccinated (*n* = 8), vaccinated (*n* = 8) and naïve unvaccinated (*n* = 8). Twenty‐four hours after the infection, colony forming units (CFU) were measured from blood and lungs **(b)**. Each symbol represents the CFU value from a single mouse; mice were considered infected (black dots) when their CFU number was higher than the limit of detection (dotted line). Empty dots represent uninfected mice with 0 recovered CFU. Statistical differences between groups are indicated by thin black lines (ns, not significant, *P < 0.05 and ***P < 0.001. Fisher's exact test, two‐tailed). **(c)** The Meso Scale Discovery (MSD) assay was used to measure the specific IgG response to seven pneumococcal capsule serotypes by quantifying their concentration (µg mL^−1^). The sera were tested in triplicate, and average values are reported. The dotted line indicates the limit of detection. The Kruskal–Wallis test followed by Dunn's multiple comparison post hoc was used for statistical analysis (ns, not significant, *P < 0.05; **P < 0.01 and ***P < 0.001).

Human vaccination with PCV uses an intramuscular (IM) route rather than the intraperitoneal route used for the mouse work described above. We therefore replicated the B‐cell depletion followed by vaccination experiment using an IM route of vaccination, also extending to 20 days between the two vaccine doses to optimize anti‐capsular antibody levels (Figure 5a). When the IM vaccinated mice were challenged using the *S. pneumoniae* pneumonia model, the results were similar to those for IP vaccinated mice (Figure 5b). The CFU recovered from the blood of infected mice confirmed reduced protection in the B‐cell–depleted group (4/8) compared to the undepleted vaccinated control (8/8), but ongoing partial protection compared to unvaccinated mice (0/8 mice protected). B‐cell–depleted mice lost the protection given by IM vaccination against lung infection, with no statistically significant difference in lung CFU to unvaccinated controls. To assess if the preserved level of protection was due to a T‐cell dependent response to the vaccine, a group of B‐cell depleted mice were also depleted of CD4^+^ T cells before *S. pneumoniae* challenge. The efficacy of the CD4^+^ T‐cell depletion was confirmed using FACS to analyse mouse splenocyte populations (Supplementary figure 2). The level of protection against *S. pneumoniae* in the combined T‐cell–depleted/ B‐cell–depleted mice level was similar to that observed for the B‐cell–depleted only group, suggesting that a different mechanism to T‐cell immunity maintained partial protection after B‐cell depletion. IgG levels against different pneumococcal capsule types were measured by MSD, showing significant differences between the vaccinated undepleted mice and the two depleted groups (Figure 5c). In particular, all the mice from either the B cell or the combined B‐ and T‐cell–depleted groups that developed infection after challenge with 6B *S. pneumoniae* did not have detectable levels of antibodies against the 6B capsule, whereas three out of nine of the mice protected against *S. pneumoniae* challenge had detectable although low levels of IgG to the 6B capsule (values measured were 0.572, 0.839 and 0.423 µg mL^−1^). These data show ongoing protection to *S. pneumoniae* in mice B‐cell depleted just before vaccination with Prevnar that was independent of T cells and perhaps mediated by low levels of specific antibody to capsule.

## Discussion

The expansion of therapeutic B‐cell depletion therapy for multiple diseases has increased the importance of obtaining data on how B cell therapies may affect both naturally and vaccine acquired immunity to important pathogens. Here we have used a preclinical mouse model to assess the effects of B‐cell depletion on Prevnar‐induced immunity to *S. pneumoniae*. The results confirmed that B‐cell depletion therapy can have an impact on vaccination induced immunity. In particular, vaccinating mice with Prevnar‐13 during periods of B cell cytopenia resulted in a markedly reduced antibody response when assessed using whole cell ELISA, flow cytometry IgG and IgM opsonization assays, and specific measurement of anti‐capsular antibody levels. However, despite these effects on vaccine responses, these mice still had substantially improved protection against *S. pneumoniae* pneumonia challenge compared to unvaccinated controls. Furthermore, B‐cell depletion after Prevnar‐13 vaccination had little effect on both Prevnar‐induced serological responses and protection against *S. pneumoniae* pneumonia.

The finding that vaccination before B‐cell depletion leads to preserved vaccine responses support clinical guidelines that suggest patients are vaccinated against a range of infectious diseases before undergoing B‐cell depletion therapy.^19^ However, if this is not possible then our data do support the use of vaccination even immediately after B‐cell depletion as 50% of mice receiving Prevnar‐13 after B‐cell depletion were still protected against septicaemia. The maintenance of some protective efficacy despite lower Prevnar‐induced antibody recognition of *S. pneumoniae* in theory could be related to preserved T‐cell immunity. However, our data showed that this was unlikely as additional T‐cell depletion did not abrogate ongoing partial protection against subsequent *S. pneumoniae* pneumonia in mice with depleted B cells prior to vaccination. This is compatible with Prevnar‐13–mediated protection generally considered to be mediated purely by antibody‐dependent mechanisms rather than antibody and T‐cell cellular responses. In addition, we have previously shown that antibody rather than T cells is essential for immunity to *S. pneumoniae* septicaemia.^23^, ^24^, ^25^ Potentially even a weak antibody response to Prevnar‐13 from the 5% of B cells surviving after B‐cell depletion treatment^26^, ^27^ is adequate to maintain some protection against septicaemia. A comparable effect to our pre‐clinical data has been recently observed for patients with multiple sclerosis, who were still capable of mounting an attenuated response to vaccines even when treated with B‐cell depletion.^28^ For our study, we have used Prevnar‐13 rather than the standard adult *S*. *pneumoniae* vaccine Pneumovax as Prevnar‐13 more consistently induces protection against *S. pneumoniae* in mouse models of infection and is also recommended for the more severely immunocompromised patients. Whether B‐cell depletion before administering Pneumovax or for vaccines to other important pathogens for adults (e.g. herpes zoster) also results in some degree of maintained protection against infection will require further evaluation.

The maintenance of both serological responses and protection against *S. pneumoniae* infection in mice B‐cell depleted after Prevnar‐13 vaccination is reassuring. Vaccine responses were maintained despite an interval of approximately twice the half‐life of IgG. It is possible that assessing Prevnar‐induced immunity after a much longer period to allow the initial vaccine‐induced antibody responses to wane could identify stronger effects of B‐cell depletion. However, the duration of an experiment assessing these effects of B‐cell depletion on recall memory responses would be impractical. IgM responses decrease more rapidly,^29^, ^30^ with a reported half‐life in blood of about 5 days compared to 23 days for IgG.^21^ Hence, loss of B cells could affect IgM‐mediated immunity at an earlier stage than IgG mediated immunity. The data for the vaccination then B‐cell depletion study did suggest this effect might be occurring as in general there were lower levels of anti–*S. pneumoniae* IgM but not IgG for these mice than the undepleted group. However, there was significant intermouse variability, and the differences observed were not statistically significant. It is unclear why there is such marked variation in antibody responses between inbred mice given identical doses of Prevnar; probably differences between mice in the functional effects of B‐cell depletion when combined with variation in immune responses due to poorly defined environmental factors could create substantial variability.

In conclusion, the B‐cell–depleted mouse model confirmed that the timing of B‐cell depletion is crucial for the efficacy of vaccination but also indicated that vaccination immediately after B‐cell depletion can still offer a degree of protection against *S. pneumoniae*. Overall, the data highlight the importance of the timing of B‐cell depletion therapy, but that vaccination even at sub‐optimal times can still have an impact in preventing *S. pneumoniae* infections. Further clinical studies on the efficacy of vaccination on B‐cell–depleted patients are needed to assess the significance of our findings in clinical practice.

## Methods

### Bacterial strains

The pneumococcal strains 6B BHN418 and TIGR4 were used for this study (capsular 6B and 4, respectively). *Streptococcus pneumoniae* was cultured in Tryptic Soy Broth (TSB, Becton Dickinson, Wokingham, UK) or grown on Columbia agar plates Agar (Becton Dickinson) supplemented with 5% v/v defibrinated horse blood at 37°C in 5% CO_2_.

### Animal models of B‐cell depletion, vaccination and infection

Five‐week‐old, female, inbred C57BL/6 mice from Charles River (Margate, Kent, UK) were used in this study. Before use, mice were housed for at least 1 week under standard conditions, in the Biological Service animal facility at the University College of London, according to its guidelines for the maintenance of laboratory animals. No randomisation or blinding was performed. All animal procedures were approved by the local ethical review process and conducted in accordance with the relevant, UK Home Office–approved, project license (PPL70/6510). B‐cell depletion on mice was induced as previously described^20^ by IP injection of aCD20 antibody (Rat IgG2b, κ, SA271G2; BioLegend, San Diego, CA, USA).^31^ The 50 µg/mouse dose has been used. Isotype control rat IgG was used as negative control. CD4^+^ T‐cell depletion has been performed administering IP 250 mg anti‐CD4 mAb (GK 1.5; BioxCell, Upper Heyford, UK) 48 and 24 h before *S. pneumoniae* challenge.^25^ For the vaccination model, mice received intraperitoneally (or intramuscularly) two doses, 10–20 days apart, of 20 µL volume of Prevnar‐13 vaccine (Pfizer, New York, NY, USA).^32^ For the pneumonia with secondary septicaemia model, mice were anaesthetized using isoflurane and then infected intranasally with pneumococcal strains 6B BHN418 using a dose of 1 × 10^7^ CFU in 50 µL volume.^25^, ^33^ Mice were culled 24 h after infection. Mouse organs were homogenized in 1 mL of PBS for quantification of colony forming units (CFU) and flow cytometry analysis. Blood samples from mice were collected by cardiac puncture under terminal anaesthesia and treated with 100 U mL^−1^ of heparin (Merck, Gillingham, UK) to prevent blood coagulation.

### Whole‐cell ELISA and flow cytometry IgG and IgM binding assays

Previously described whole‐cell ELISAs and flow cytometry assays^25^ have been used to assess antibody recognition of *S. pneumoniae*. For both assays each serum have been tested in triplicate. Briefly, for whole‐cell ELISAs, *S. pneumoniae* were grown to an OD_600_ of approximately 0.4–0.8, washed and resuspended to an OD_600_ of 0.4 in PBS; 50 μL well^−1^ were added to microtitre plates and incubated overnight at RT before fixation in 4% paraformaldehyde for 10 min. Plates were washed and incubated with a 1:100 dilution of murine antiserum for 1 h at 37°C and using HRP‐conjugated goat anti‐mouse IgG (ab6789; abCam, Cambridge, UK) for detection. For flow cytometry antibody binding assays live *S. pneumoniae* (1 × 10^6^ CFU) were incubated for 30 min at 37°C with 10% mouse serum. Fluorescently labelled anti‐mouse IgG (FITC, 405308; BioLegend) and IgM (APC, 406505; BioLegend) were used to detect antibody binding to the bacterial surface using a Becton Dickinson FACSVerse instrument.

### Splenocytes isolation and analysis

Splenocytes were prepared by passing mouse spleens through a cell strainer to obtain single cell suspensions; red blood cells were removed using a red blood cell lysis buffer (BioLegend, 420301). For the B cell reconstitution experiment (Figure 2b) splenocytes were stained using fluorescently conjugated antibodies to define B and T cells populations using the following surface markers: CD19 (B cells; BioLegend, 115529) and CD3 (T cells; BioLegend, 100217). Dead cells were excluded using a viability die (Zombie Green Fixable Viability Kit; BioLegend, 423111). In the T‐cell depletion experiment instead, splenocytes have been stained with the following antibodies: CD19 (BioLegend, 152409), CD3 (BioLegend, 362703), CD4 (BioLegend, 100438), CD8 (BioLegend, 155004), CD11b (BioLegend, 101245), CD11c (BioLegend, 117317), and Ly‐6G (BioLegend, 1527615). The full gating strategy is reported in Supplementary figure 1. Dead cells were excluded using a viability die (Zombie Green Fixable Viability Kit; BioLegend, 423111). Samples have been analyzed using a Becton Dickinson FACSVerse, and data have been processed using FlowJo software for Windows (Becton Dickinson, Franklin Lakes, New Jersey, USA, version 10).

### Measurement of IgG recognition of specific *S. pneumoniae* capsular polysaccharides

Antibody levels to selected *S. pneumoniae* capsular antigens (5 μg mL^−1^) were measured using a multiplexed electroluminescence assay as previously described^23^, ^34^ using a Meso Scale Discovery (MSD; Rockville, MD, USA) platform assay. After incubation of each antigen‐coated plate with blocking agent, washing, and incubation with diluted test serum for 45 min at room temperature, the plates were washed and MSD assay sulfo tag–labeled goat anti‐mouse IgG secondary antibody (MSD, R32AC‐1) was added for reading using an MSD Sector Imager 2400 or 6000 apparatus.

### Statistical analysis

Statistical analyses were conducted using Prism 8 (Graph Pad, San Diego, CA, USA). Unless otherwise stated, data are presented as means, and error bars represent standard deviations. Due to significant variations between individual mice combined with the relatively small sample sizes the data were analysed using non‐parametric tests. The Kruskal–Wallis test followed by Dunn's multiple comparison post hoc was used to compare ELISA, MSD and serum deposition assay data. When mouse CFU data included a large number of mice with zero recovered CFU, they were analysed with Fisher’s exact test to compare the number of infected versus uninfected animals for each group rather than mean or median CFU.

## Conflict of Interest

GW was employed by the company Novartis Institute for BioMedical Research. The remaining authors declare that the research was conducted in the absence of any commercial or financial relationships that could be construed as a potential conflict of interest.

## Author Contributions

**Giuseppe Ercoli:** Conceptualization; Data curation; Formal analysis; Investigation; Methodology; Visualization; Writing – original draft; Writing – review & editing. **Elisa Ramos‐Sevillano:** Data curation; Investigation; Methodology. **Emma Pearce:** Investigation; Methodology. **Sara Ragab:** Investigation; Methodology. **David Goldblatt:** Investigation; Methodology; Resources. **Gisbert Weckbecker:** Conceptualization; Funding acquisition; Resources. **Jeremy Brown:** Conceptualization; Data curation; Funding acquisition; Project administration; Supervision; Writing – review & editing.
