# Supplementary material for: Maintained partial protection against Streptococcus pneumoniae despite B‐cell depletion in mice vaccinated with a pneumococcal glycoconjugate vaccine
Source: Clin Transl Immunology. 2021 Dec 28;11(1):e1366. doi: 10.1002/cti2.1366 (PMC8715227; doi:10.1002/cti2.1366)
Supplement: Supplementary file 1 [file CTI2-11-e1366-s001.pdf]

Supporting information

Maintained partial protection against *Streptococcus pneumoniae* despite B cell depletion in mice vaccinated with a pneumococcal glycoconjugate vaccine

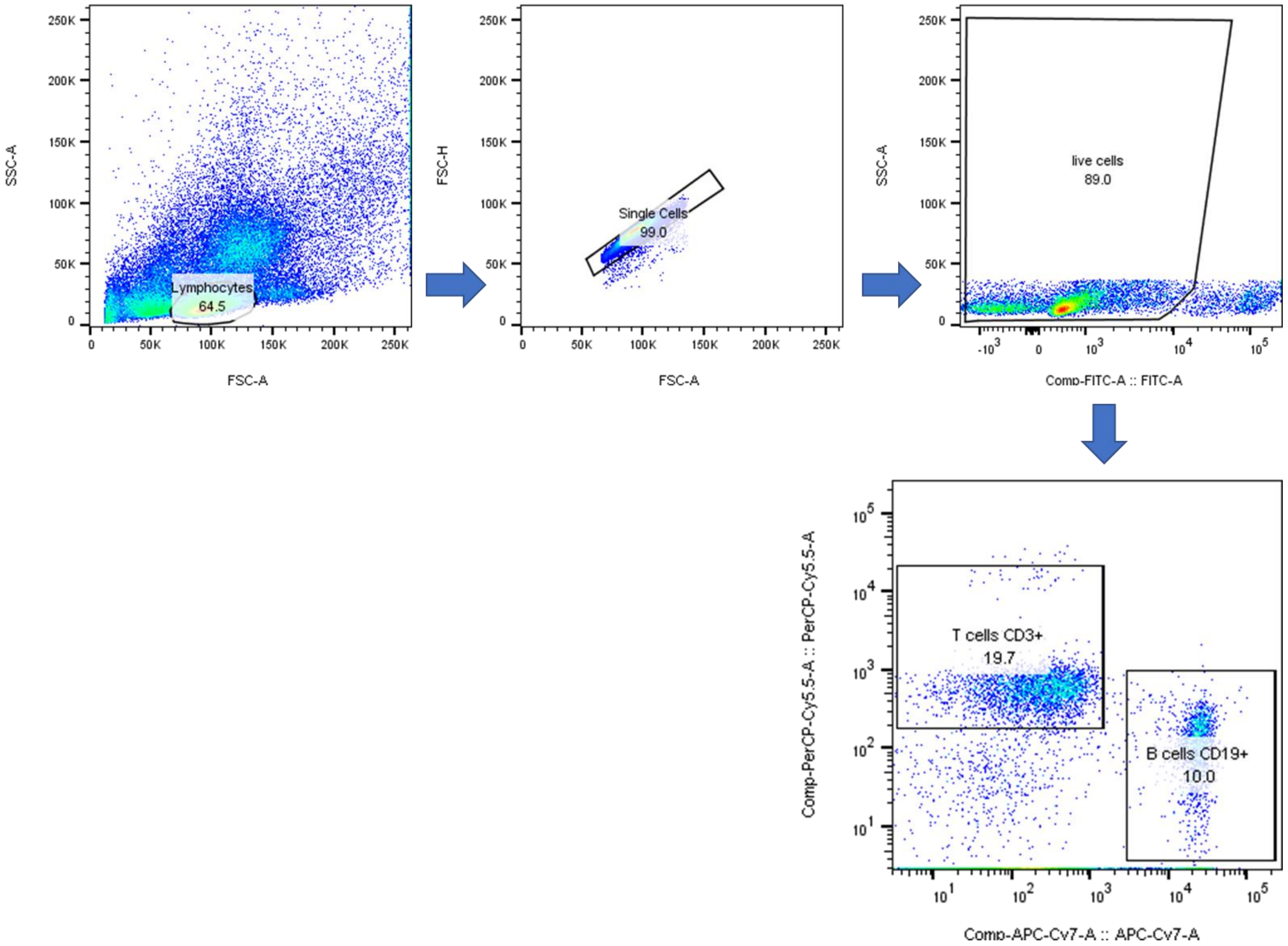

Supplementary figure 1. FACS gating strategy B cell repopulation.

Splenocytes have been analysed with BD FACS Verse. Firstly, the lymphocytes population has been identified based on its size, then single cells have been selected plotting FSC-H vs FSC-A. A viability dye has been used to discard all the dead cells and finally the B and T cells populations have been identified based on the presence of the CD19 and CD3 surface markers respectively.

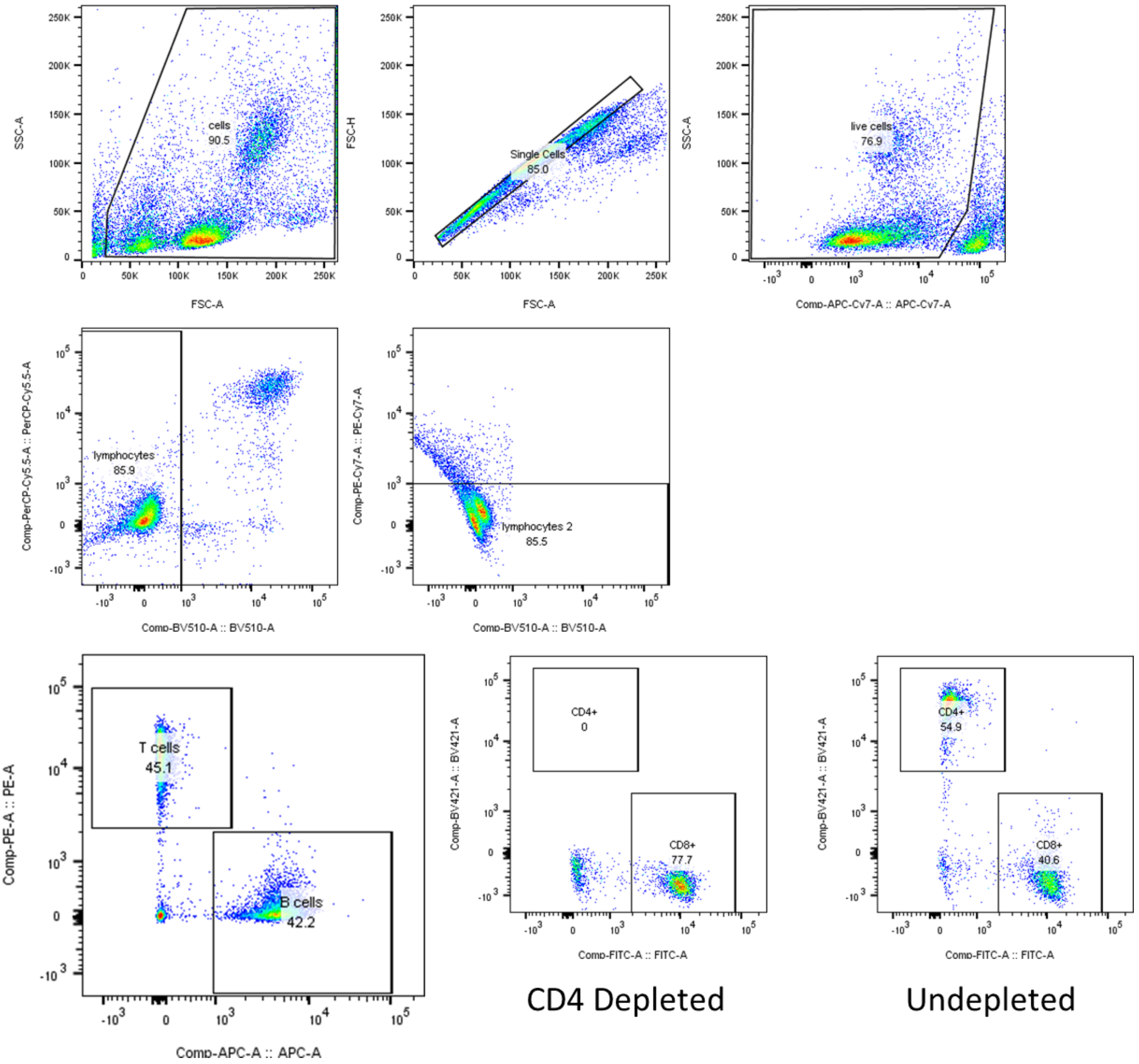

Supplementary figure 2. FACS gating strategy T cell depletion (Fig.5).

Splenocytes have been analysed with BD FACS Verse. Firstly, single cells have been selected plotting FSC-H vs FSC-A. A viability dye has been used to discard all the dead cells, all cells that were CD11b+LY-6G+ (macrophages) and CD11c+ (dendritic cells, monocytes) have been also excluded to analyse only the lymphocytes population. B and T cells populations have been identified based on the presence of the CD19 and CD3 surface markers respectively. CD3+ cells have been further divided into CD4+ and CD8+. Absence of CD4+ cells has been confirmed in all CD4 depleted mice.
